# Caveolin-1 induces lamellipodia formation via an Akt-dependent pathway

**DOI:** 10.1186/1475-2867-14-52

**Published:** 2014-06-14

**Authors:** Pithi Chanvorachote, Preedakorn Chunhacha, Varisa Pongrakhananon

**Affiliations:** 1 Department of Pharmacology and Physiology, Faculty of Pharmaceutical Sciences and Cell-based Drug and Health Product Development Research Unit, Chulalongkorn University, Pathumwan, Bangkok 10330, Thailand

**Keywords:** Lamellipodia, Cancer migration, Lung cancer, Caveolin-1

## Abstract

**Background:**

The enhancement of migration is critical for facilitating cancer cell metastasis.

**Method:**

Lung cancer H23 cells were transfected with either a caveolin-1 (Cav-1) overexpression or shCav-1 plasmid and further subjected to cell migration assays and lamellipodia characterization. The regulation of Cav-1 via an ATP-dependent tyrosine kinase (Akt) pathway was further examined by Akt knockdown in Cav-1 overexpressing cells and migratory behavior investigations.

**Results:**

Here, we demonstrate for the first time that overexpression of Cav-1 in human lung cancer H23 cells significantly increased the formation of lamellipodia, whereas the suppression of Cav-1 using shRNA transfection had the opposite effect. Consistent with an increase in lamellipodia, Cav-1 overexpressing cells exhibited increased migratory activity in comparison to their parental, control-transfected, H23 cells. The induction of lamellipodia was demonstrated to occur via the Akt pathway because the addition of the Akt inhibitor LY294002 inhibited lamellipodia in both Cav-1-overexpressing and H23 cells. Additionally, transient transfection with Akt-siRNA significantly inhibited the formation of lamellipodia and the migration of Cav-1-overexpressing H23 cells. In addition, Cav-1 levels and the migratory action of other lung cancer cells, namely, H460 and A549, were assessed, and the migration of these cells was found to be correlated with the basal Cav-1 level.

**Conclusion:**

These data showed that Cav-1 enhances cancer cell migration through Akt-mediated lamellipodia formation. Our results provide novel insights regarding the molecular mechanism controlling cancer cell migration, leading to a better understanding of cancer cell biology.

## Background

Understanding the molecular mechanisms that control cancer cell behavior is important for the development of novel anti-cancer strategies. In lung cancer, an enhanced migration ability of the cells is among the key factors facilitating metastasis, a major cause of death in this type of cancer. Therefore, strategies that inhibit or attenuate the process of cancer dissemination, including migration, have garnered much research attention in the field.

Lamellipodia are widely accepted to be critical for directional migration in many cells [1]. In cancer, an increased amount of lamellipodia is frequently found in highly motile cells, and lamellipodia are believed to play a key role in potentiating cancer motility and metastasis [2]. Indeed, cell migration is initiated by the formation of pseudopodia such as lamellipodia, sheet-like cellular protrusions that are enriched with F-actin. The formation of lamellipodia involves multi-step processes of actin polymerization and depolymerization [3]; actin filaments are arranged into a sheet-like network of lamellipodia at the leading edge of the cells during movement [3]. The defined molecular pathways controlling the formation of lamellipodia have been elusive to date, and such insight is considerably vital to the discovery of novel molecular targets in the development of anti-metastasis therapies.

Caveolin-1 (Cav-1), a protein comprising the portions of membranes called caveolae, was previously shown to have a potentiating effect on the progression of cancers [4-7]. In addition, the expression level of Cav-1 was shown to be associated with a poor prognosis and metastasis in several cancers, including prostate [8], pancreas [9], and lung cancers [10]. In particular, Cav-1 was reported to be a key regulator of anoikis resistance [4-6] and migration and invasion [7]; however, its regulatory role in controlling lamellipodia and the possible regulatory mechanism are largely unknown. Therefore, we aimed to investigate the possible impact of the Cav-1 protein on lamellipodia formation and cancer cell motility in human lung cancer cells. For the first time, we show that Cav-1 induces the formation of lamellipodia and increases tumor cell motility through an Akt-dependent mechanism. Our study suggests the novel hypothesis that the Cav-1 protein has a positive regulatory function in the process of cancer cell metastasis.

## Results

### Caveolin-1 enhances lamellipodia formation and migration in non-small cell lung cancer H23 cells

**Figure 1:**
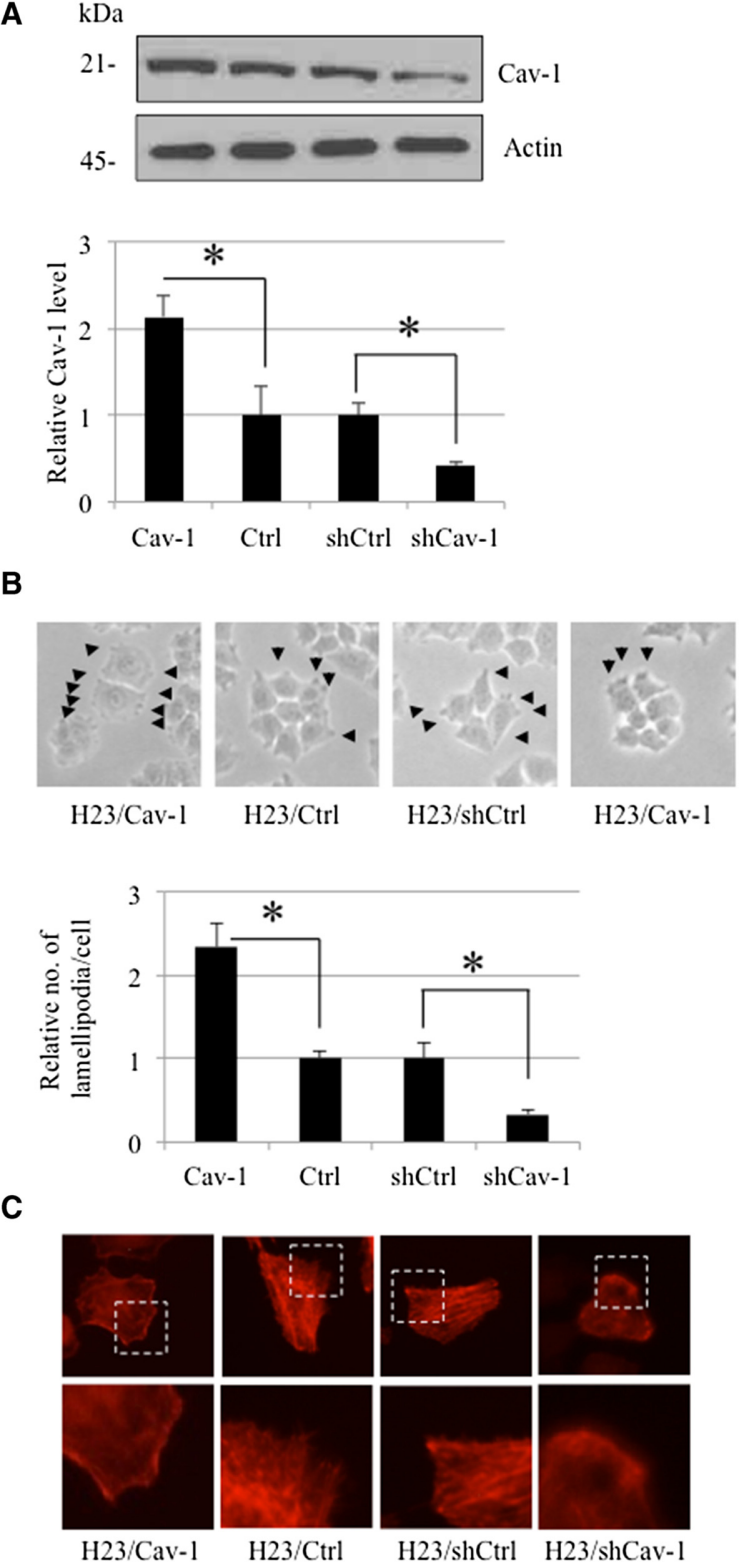
**Caveolin-1 enhances lamellipodia formation in lung cancer H23 cells. (A)** Cells were transfected with Cav-1 overexpressing, control, shCav-1 or shRNA scrambled plasmids and analyzed for Cav-1 level by western blotting. The blots were reprobed with β-actin to confirm equal loading. The immunoblot signals were quantified by densitometry, and mean data from independent experiments were normalized to the results. The bars are the means ± SD (n = 4). *, p < 0.05 vs. control (H23/Ctrl) or shRNA scrambled (H23/shCtrl) transfected cells. **(B)** Lamellipodia in the transfected cells was indicated by arrow. Data represent as relative number of lamellipodia/cell as described in Material and Methods. Plots are mean ± SD (n = 4). *, p < 0.05 vs. control (H23/Ctrl) or shRNA scrambled (H23/shCtrl) transfected cells. Lamellipodia at the edge of cells were indicated by arrow. **(C)** Transfected cells were stained with phalloidin-rhodamine and visualized under fluorescent microscope.

To test the effect of Cav-1 protein in the regulation of lamellipodia formation, we stably transfected H23 cells with Cav-1-overexpression, control, shRNA-Cav-1 or shCtrl plasmids and selected stable transfectants using an appropriate protocol. The Cav-1 overexpressing (H23/Cav-1), H23 control (H23/Ctrl), Cav-1 knockdown (H23/shCav-1) and shRNA control (H23/shCtrl) cells were analyzed for Cav-1 levels using western blotting, as described in Materials and Methods. Figure 1A shows that the cells transfected with the Cav-1 overexpression plasmid exhibited a higher level of the protein in comparison to that of the control cells. In contrast, the shCav-1 transfectants had the lowest level of Cav-1.Furthermore, these cells were analyzed for the formation of cell protrusions under inverted light microscopy and fluorescence microscopy. Figure 1B and C indicate that the Cav-1-overexpressing cells exhibited a significant increase in the number of sheet-like lamellipodia in comparison to that of the parental H23 cells, whereas the Cav-1-knockdown cells displayed the fewest number of lamellipodia. These results demonstrate for the first time that Cav-1 plays a positive role in the formation of cellular lamellipodia in lung cancer cells.

### Lamellipodia enhances H23 cell migratory activity

**Figure 2:**
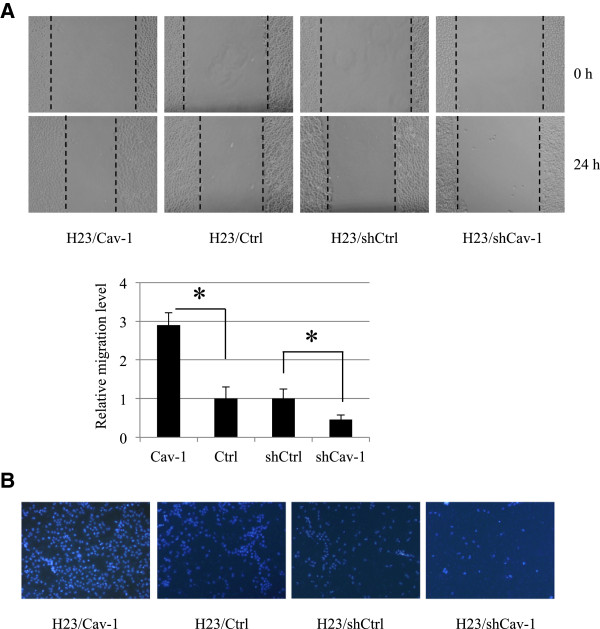
**Caveolin-1 increases lung cancer H23 cell migration. (A)** Confluent monolayers of Cav-1 transfected (H23/Cav-1), control (H23/Ctrl), shRNA-Cav-1-transfected (H23/shCav-1), and shRNA scrambled (H23/shCtrl) cells were wounded using a 1-mm-wide tip and allowed to migrate for 24 h. The wound space was visualized under a microscope and represented as relative migration level. Data are means ± SD (n = 4). *, p < 0.05 vs. control transfected cells. **(B)** For transwell assay, cell migration was examined by a transwell assay for 24 h. Migratory cells at the basolateral side of the membrane were stained with Hoechst 33342 for 30 min and visualized under a fluorescence microscope.

Because lamellipodia were linked to the migratory activity of the cells, we further tested whether such presented lamellipodia are associated with cell migration. Figure 2 shows that Cav-1-overexpressing H23 cells exhibited the dominant migratory activity across a wound space; the shCav-1-transfected cells showed the opposite behavior. Additionally, the relative migration level obtained from Transwell assays, as described in Materials and Methods, indicated a similar trend that the cells possessing a higher level of Cav-1 migrate faster than the control group and shRNA-Cav-1-transfected cells. These data are consistent with previous reports that lamellipodia in cancers are associated with cell motility. Our results reveal the positive role of Cav-1 on lamellipodia formation and cancer cell migration.

### Caveolin-1 enhances lamellipodia via an Akt-dependent mechanism

**Figure 3:**
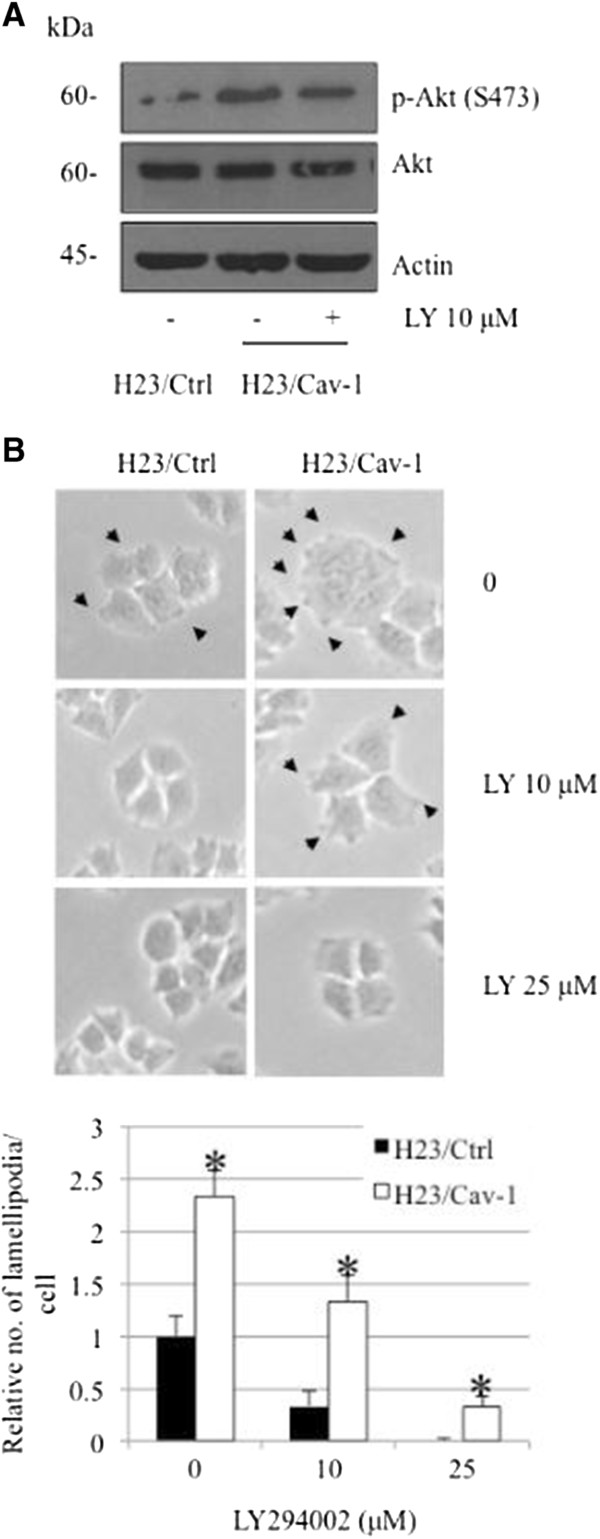
**Effect of Akt inhibitor on lamellipodia formation. (A)** Cav-1 overexpressed (H23/Cav-1) and control (H23/Ctrl) H23 cells were treated with Akt inhibitor (LY294002) at the concentration of 10 μM. Expression level of total Akt and activated Akt was determined. The blots were reprobed with β-actin to confirm equal loading. **(B)** Lamellipodia in control (H23/Ctrl) and Cav-1 overexpressing (H23/Cav-1) cells were indicated by arrow. Data were plotted as relative number of lamellipodia per cell in each field and represented the means ± SD (n = 4). *, *p* < 0.05 vs. control transfected (H23/Ctrl) cells at each treatment.

**Figure 4:**
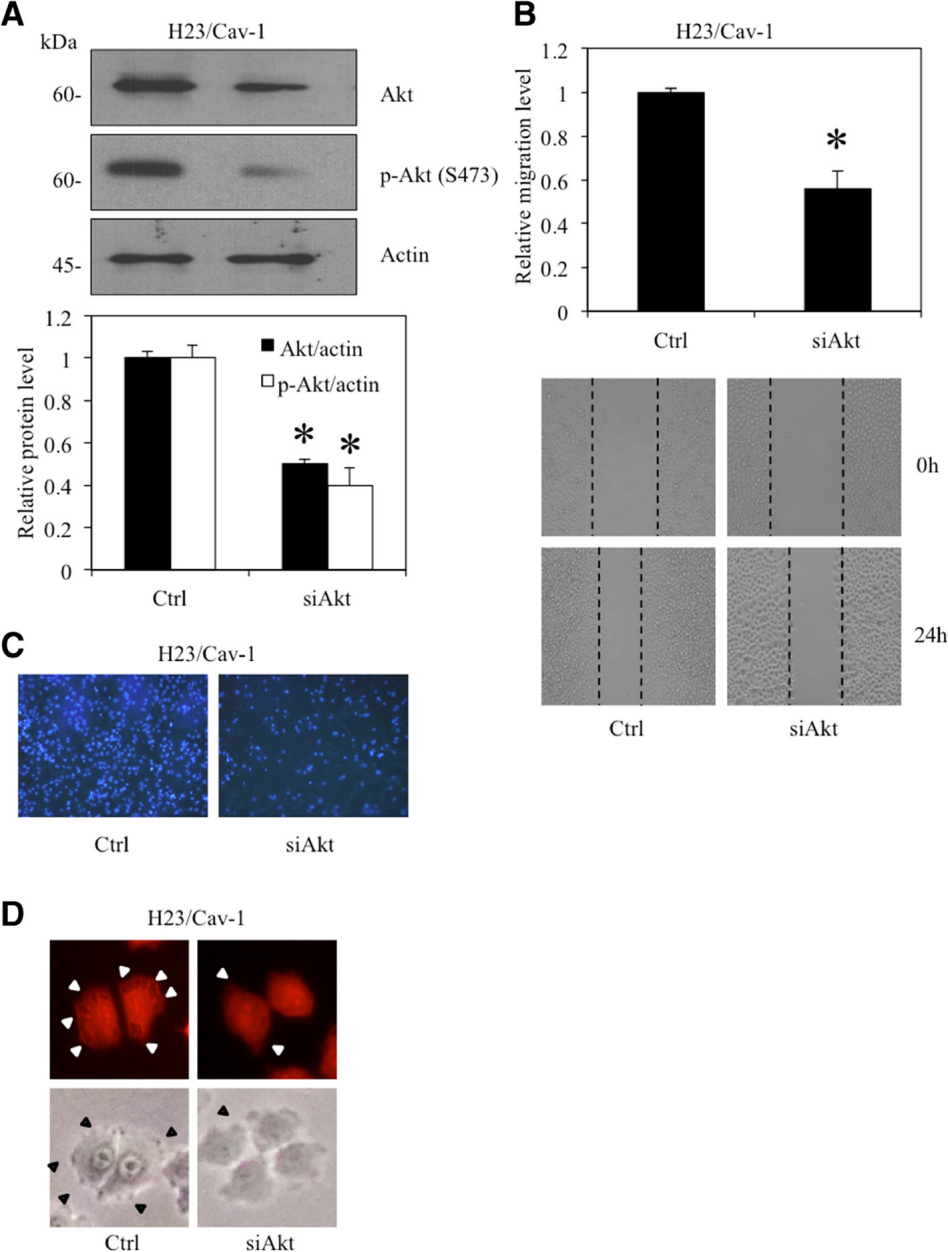
**Caveolin-1 regulates lamellipodia via Akt-dependent mechanism.** Cav-1 overexpressed (H23/Cav-1) cells were transfected with siRNA-Akt. **(A)** Akt and activated Akt levels were analyzed by western blot analysis. The immunoblot signals were quantified by densitometry, and mean data from independent experiments were normalized to the results. The bars are the means ± SD (n = 4). *, *p* < 0.05 vs. control transfected (H23/Cav-1) cells. **(B)** Migratory activity of H23/Cav-1 transfected with siRNA-Akt was determined by wound healing assay. Data represent the means ± SD (n = 4). *, p < 0.05 vs. control transfected (H23/Cav-1) cells. **(C)** Migratory activity of Cav-1 overexpressing cells transfected with either siAkt or control plasmid were investigated by transwell migration assay as described. **(D)** Lamellipodia in the cells were investigated and indicated by arrow as described.

Akt was shown to play a critical role in mediating cancer cell migration through the induction of actin polymerization [11,12]. To provide the possible underlying mechanism of the inductive effect of Cav-1 on lamellipodia in these cells, we tested whether Cav-1 up-regulates lamellipodia in an Akt-dependent mechanism. The Cav-1-overexpressing and control H23 cells were subjected to a western blot analysis to assess the level of activated Akt and total Akt. The results indicated that the Cav-1-overexpressing cells showed an increased level of activated Akt (phosphorylated Akt at Ser473), whereas the level of total Akt was comparable to that of the control H23 cells (Figure 3A).In addition, we tested whether such up-regulation of activated Akt plays a role in controlling lamellipodia formation in these cells. Cells overexpressing Cav-1 and control H23 cells were treated with LY294002, a selective PI3K/Akt inhibitor, for 24 h, and lamellipodia were examined as described. Figure 3B shows that treatment with the Akt inhibitor significantly inhibited lamellipodia formation in these cells, consistent with a suppression of Akt activation (Figure 3A). These data indicate that Cav-1 enhances lamellipodia in cells via Akt up-regulation.To confirm the role of Akt in Cav-1-lamellipodia regulation in these cells, Cav-1-overexpressing cells were transiently transfected with siRNA-Akt, and lamellipodia were analyzed. Western blotting revealed that both total and phosphorylated Akt in the cells transfected with siRNA-Akt were significantly decreased (Figure 4A). Additionally, the migratory activity of the H23/Cav-1 cells was significantly suppressed by the transfection with the siRNA targeting Akt (Figure 4B and C). In accordance with the above finding, we found that the siRNA transfectants exhibited dramatically low levels of lamellipodia per cell (Figure 4D). Taken together, these results reveal a novel molecular pathway in the regulation of cancer cell migration via Cav-1 and Akt pathways.

### The endogenous Caveolin-1 level correlates with human lung cancer cell migratory behavior

**Figure 5:**
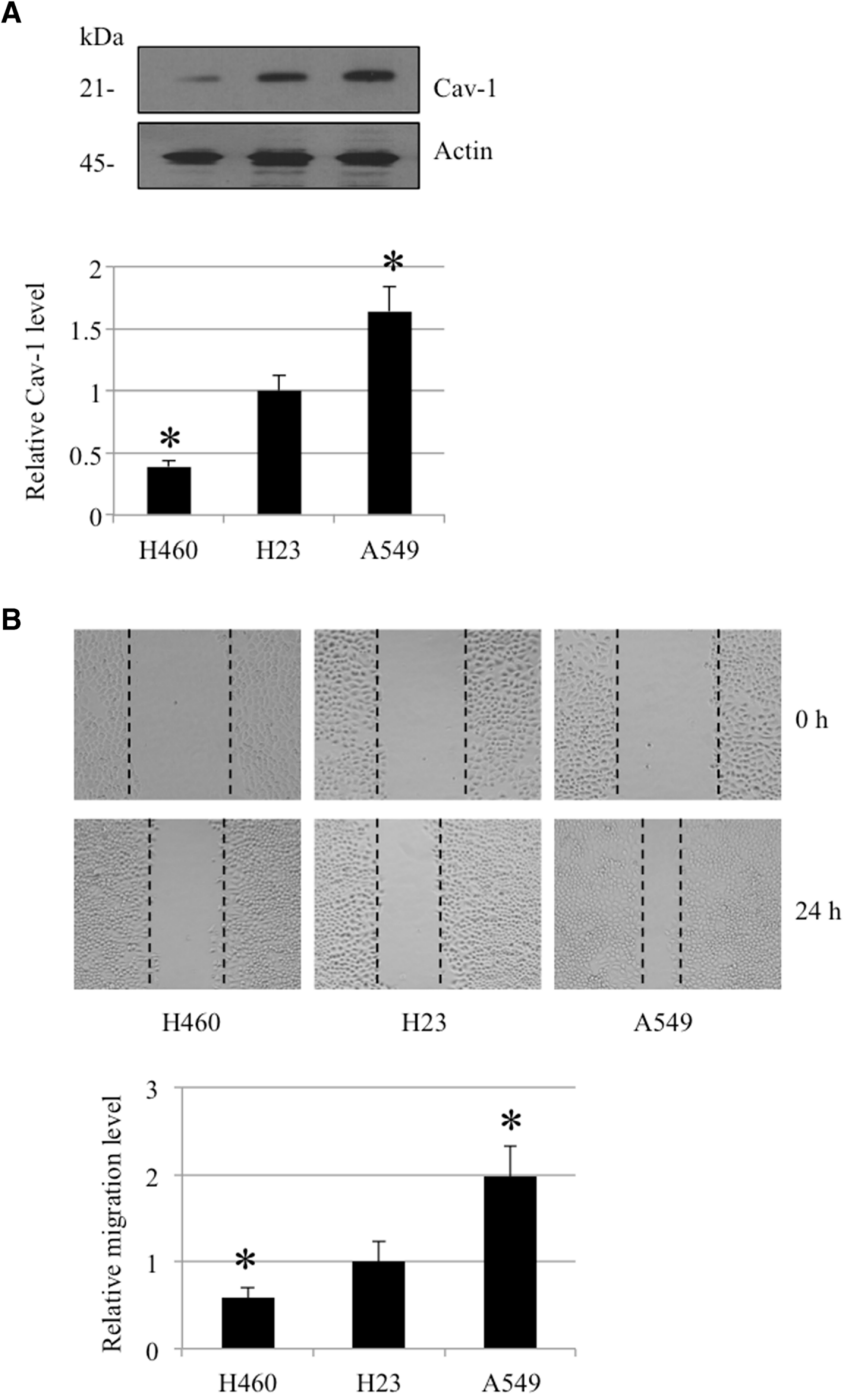
**Basal level of Caveolin-1 in lung cancer cells correlates with their migratory potential. (A)** H460, H23, and A549 were analyzed for endogenous Cav-1 level by western blot analysis. **(B)** Migration of all cells was determined as described in Materials and Methods. Data represent the means ± SD (n = 4). *p < 0.05 vs. H23 cells.

Others and we have reported the potentiating action of Cav-1 on cancer aggressiveness and metastasis [4-10]. Thus, we assessed whether the basal expression level of Cav-1 in human lung cancer cells could affect their migration. Human lung cancer cells, namely, H460, H23 and A549 cells, were analyzed for endogenous Cav-1 levels by a western blot analysis. Additionally, the migratory activity of the cells was determined by a wound-healing assay. Figure 5 shows that A549 cells, which possess the highest level of Cav-1 protein among the tested cells, exhibited the highest degree of migration. In contrast, H460 cells, which had the lowest basal Cav-1 level, showed the least migration. These results support the above finding that Cav-1 has a positive role in the regulation of lung cancer cell migration.

## Discussion and Conclusion

The migratory activity of cancer cells is generally recognized as an important factor for successful metastasis. In lung cancer, the high mortality rate is caused by cancer metastasis, which in this type of cancer is frequently found at the time of diagnosis [13]. Cancer researchers have paid much attention to identifying possible strategies for inhibiting cancer migration, and knowledge of the molecular basis of such cell activity is highly critical.

Cav-1 is a major protein component of the cell structures called caveolae. This protein has lately garnered a significant amount of attention in cancer research because a large number of studies have indicated its role in potentiating cancer cells in many ways [14-16]. Recently, we reported a role for Cav-1 in the regulation of anoikis [4-6], migration and invasion [7] in a lung cancer cell model; however, its role in controlling lamellipodia is still unknown. The migration of cancer cells involves several regulatory proteins, and among them Akt plays a central role. An increase in cellular activated Akt is observed in metastatic tumors, and activated Akt has been shown to increase cell motility [17,18]. Akt contributes to the polymerization of actin protein, resulting in the cell protrusions called lamellipodia. Here, we reveal for the first time that Cav-1 has a function in controlling the lamellipodia formation that leads to cell migration. The number of lamellipodia per cell has been correlated with an increased migratory behavior of cancer cells [2,19,20]. Our results are consistent, as we found that both lamellipodia and migration significantly increased in Cav-1-overexpressing cells (Figures 1 and 2). Additionally, shRNA transfection confirmed this function of Cav-1 by causing the opposite effect.To provide a molecular mechanism by which Cav-1 regulates lamellipodia, an Akt inhibitor as well as siRNA-Akt were used. We found that the decrease in activated Akt caused by the Akt inhibitor or siRNA transfection was able to abolish the effect of Cav-1 (Figures 3 and 4), suggesting that Cav-1 up-regulates lamellipodia via an Akt-dependent mechanism. Because the up-regulation Cav-1 was demonstrated in tumor tissues from patients and the high expression level of this protein was linked to a poor prognosis, we hypothesized that the basal expression level of Cav-1 protein may affect the migratory activity of lung cancer cells. Human lung cancer cells, including H460 and A549 cells, were subjected to a western blot analysis, and our results showed that the cells possessing a higher level of Cav-1 exhibited greater motility (Figure 5). These observations support the results of our experiments that an increase in cellular Cav-1 contributes to an increase in cell motility. Although the migration activity of lung cancer cells is likely to involve several pathways, our results and observations indicate that Cav-1, at least in part, plays a critical role in the regulation of lung cancer cell migration.

In conclusion, the present study reveals information regarding an endogenous protein that plays a significant role in cancer cell behavior. This insight may provide a better understanding of lung cancer and lead to the development of novel therapies.

## Materials and Methods

### Cells and reagents

The non-small cell lung cancer (NSCLC) cell lines H23, A549 and H460 were obtained from the American Type Culture Collection (Manassas, VA). The cells were cultured in RPMI-1640 supplemented with 5% fetal bovine serum (FBS), 2 mM L-glutamine and 100 units/mL penicillin/streptomycin. The cells were incubated in a 5% CO_2_ environment at 37°C. Phalloidin tetramethylrhodamine B isothiocyanate and LY294002 were obtained from Sigma Chemical, Inc. (St. Louis, MO). Antibodies against pan-Akt, p473-Akt, FAK, caveolin-1 and β-actin and peroxidase-conjugated secondary antibodies were obtained from Cell Signaling Technology, Inc. (Danvers, MA).

### Migration assay

Migration was determined by wound-healing and Transwell assays. For the wound-healing assay, a monolayer of cells was cultured in a 24-well plate, and a wound was achieved using a 1-mm-wide pipette tip. The cell monolayers were allowed to migrate for 24 h. Micrographs were taken under a phase-contrast microscope (Olympus DP70, Melville, NY), and the wound spaces were measured from 10 random fields of view using Olympus DP controller software. A quantitative analysis of cell migration was performed using an average wound space from those random fields of view, and the percentage of change in the wound space was calculated using the following formula: % change = (average space at time 0 h) - (average space at time 24 h)/(average space at time 0 h) × 100. For the Transwell assay, the cells were seeded in serum-free medium at a density of 5 × 10^4^ cells/well in the upper chamber of a Transwell device (8 μm pore size) in a 24-well plate and incubated for 24 h. RPMI medium containing 10% FBS was added to the lower chamber. Following the incubation, the non-migrated cells in the upper chamber were removed by wiping with a cotton swab, and the cells that migrated to the underside of the membrane were stained with 10 μg/mL Hoechst 33342 for 10 min, visualized and scored under a fluorescence microscope (Olympus IX51 with DP70).

### Cell morphology and lamellipodia characterization

Cell morphology was investigated by a phalloidin-rhodamine staining assay. The cells were seeded at a density of 5×10^3^ cells/well in a 96-well plate overnight. The cells were washed with PBS, fixed with 4% paraformaldehyde in PBS for 10 min at 37*°*C, permeabilized with 0.1% Triton-X100 in PBS for 4 min and blocked with 0.2% BSA for 30 min. Afterwards, the cells were incubated with 1:100 phalloidin-rhodamine in PBS for 15 min, rinsed 3 times with PBS and mounted with 50% glycerol. Cell morphology was assessed by fluorescent imaging (Olympus IX51 with DP70). The lamellipodia were calculated from an average number of flat sheet-like structures per cell, counting all of the cells present in the field (at least 50 cells/field), and represented as a number relative to the control [21,22]. At least 5 random fields were captured per sample, and four independent experiments were performed.

### Plasmids and transfection

The Cav-1 expression plasmid, Cav-1 knockdown shRNA-Cav-1 plasmid and control empty and shRNA scrambled vectors were obtained from Santa Cruz Biotechnology (Santa Cruz, CA). Stable transfections of the Cav-1 expression plasmid or Cav-1 knockdown plasmid were generated by culturing H23 cells in a 6-well plate until they reached 60% confluence. Lipofectamine reagent (15 ml) and 2 mg of Cav-1 or shRNA-Cav-1 plasmid were used to transfect the cells in the absence of serum. After 12 h, the medium was replaced with a culture medium containing 5% fetal bovine serum. Approximately 36 h after the beginning of the transfection, the cells were digested with 0.03% trypsin; the cell suspensions were seeded in 75-ml culture flasks and cultured for 24 to 28 days with drug selection. The stable transfectants were pooled, and the expression of Cav-1 protein in the transfectants was confirmed by western blotting. The cells were cultured in antibiotic-free RPMI-1640 medium for at least two passages before use in each experiment. For transient Akt knockdown, 15 ml of Lipofectamine reagent and 2 mg of either siAkt or control plasmid were mixed and applied to 60% confluent cavolin-1-overexpressing (H23/Cav-1) cells. After 12 h, the medium was replaced with a culture medium containing 5% fetal bovine serum. The transfected cells were used for experiments at approximately 36 h after the beginning of the transfection.

### Western blotting

Cells were incubated in lysis buffer containing 20 mM Tris–HCl (pH 7.5), 1% Triton X-100, 150 mM sodium chloride, 10% glycerol, 1 mM sodium orthovanadate, 50 mM sodium fluoride, 100 mM phenylmethylsulfonyl fluoride and a commercial protease inhibitor cocktail (Roche Molecular Biochemicals) for 30 min on ice. The cell lysates were collected, and the protein content was determined using the Bradford method (Bio-Rad Laboratories, Hercules, CA). Equal amounts of protein from each sample (60 μg) were denatured by heating at 95°C for 5 min in Laemmli loading buffer and were subsequently loaded onto a 10% SDS-polyacrylamide gel. After separation, the proteins were transferred onto 0.45-μm nitrocellulose membranes (Bio-Rad). The membranes were blocked for 1 h in 5% nonfat dry milk in TBST (25 mM Tris–HCl (pH 7.5), 125 mM NaCl and 0.05% Tween 20) and incubated overnight with the appropriate primary antibodies at 4°C. The membranes were washed twice with TBST for 10 min and incubated with horseradish peroxidase-coupled isotype-specific secondary antibodies for 1 h at room temperature. The immune complexes were detected by enhancement with a chemiluminescence substrate (Supersignal West Pico; Pierce) and quantified using analyst/PC densitometry software (Bio-Rad).

### Statistical analysis

The mean data from independent experiments were normalized to the results for the cells in the control group. All of the experiments were repeated at least four times. A statistical analysis between two groups was verified by Student’s t-test; to compare multiple groups, an analysis of variance (ANOVA) with a post-hoc test was conducted. A *p*-value of less than 0.05 was considered statistically significant.

## Abbreviations

Akt: ATP-dependent tyrosine kinase; PBS: phosphate-buffered saline; p-Akt: phosphorylated-Akt; Cav-1: caveolin-1; TBST: Tris-buffered saline containing Tween.

## Competing interests

The authors declare that they have no competing interests.

## Authors’ contribution

PC and VP carried out the experiments. PC, PC and VP participated in the design of the study and performed the statistical analysis. PC, PC and VP conceived of the study, and participated in its design and coordination and helped to draft the manuscript. All authors read and approved the final manuscript.
